# An Evaluation of the Nutritional Value and Physical Properties of Blenderised Enteral Nutrition Formula: A Systematic Review and Meta-Analysis

**DOI:** 10.3390/nu12061840

**Published:** 2020-06-20

**Authors:** Omorogieva Ojo, Amanda Rodrigues Amorim Adegboye, Osarhumwese Osaretin Ojo, Xiaohua Wang, Joanne Brooke

**Affiliations:** 1 School of Health Sciences, Faculty of Education, Health and Human Sciences, University of Greenwich, Avery Hill Campus, Avery Hill Road, London SE9 2UG, UK; 2 School of Human Sciences, Faculty of Education, Health and Human Sciences, University of Greenwich, Greenwich Campus, London SE10 9LS, UK; A.Adegboye@greenwich.ac.uk; 3 South London and Maudsley NHS Foundation Trust, University Hospital, Lewisham High Street, London SE13 6LH, UK; Osarhumwese.Ojo@slam.nhs.uk; 4 The School of Nursing, Soochow University, Suzhou 215006, China; wangxiaohua@suda.edu.cn; 5 Faculty of Health, Education and Life Sciences, Ravensbury House, Birmingham City University, City South Campus, Birmingham B15 3TN, UK; joanne.brooke@bcu.ac.uk

**Keywords:** enteral nutrition formula, blended formula, commercial feed, blenderised enteral formula, blenderised tube feeding, enteral tube feeding, nutritional value, physical properties of food

## Abstract

Background: Although there are merits in using commercial “enteral nutrition formula” (ENF) compared with blended ENF, there is a growing preference for the use of blended ENF in many countries globally. However, the nutritional value and physical properties of blended ENF compared with commercial ENF may be limiting its use. We have not found any evidence of a meta-analysis on the nutritional value of blended diets in the adult population. Aim: The aim of this review was to compare the nutritional value, physical properties, and clinical outcomes of blended ENF with commercial ENF. Methods: The preferred reporting items for systematic reviews and meta-analyses were used for this review. The search strategy was based on a Population, Intervention, Comparator, Outcome framework. The following databases; Pubmed, EMBASE, PSYCInfo, and Google scholar were searched for articles of interest using keywords, Medical Subject Heading (MeSH) and Boolean operators (AND/OR) from the inception of each database until 23 February 2020. The articles were evaluated for quality. Results: Based on the systematic review and meta-analysis, four distinct themes were identified; Nutritional value, Physical properties, Clinical outcomes; and Adverse events. The findings of this review showed inconsistencies in the macronutrient and micronutrient values of the blenderised ENF compared with the commercial ENF. The results of the meta-analysis demonstrated that there were no significant differences (*p* > 0.05) between the blenderised ENF and the commercial ENF in relation to the fat and protein contents of the diets. However, the blenderised ENF was significantly lower (*p* < 0.05) than the commercial ENF regarding the energy content of the diets, with an overall mean difference of −29.17 Kcal/100 mL (95% CI, −51.12, −7.22) and carbohydrate content with an overall mean difference of -5.32 g/100 mL (95% CI, −7.64, −3.00). In terms of sodium, potassium, and vitamin A, there were no significant differences (*p* > 0.05) between the blenderised and commercial ENF, although significant differences (*p* < 0.05) were observed between the two diets with respect to calcium, phosphorus, magnesium, zinc, iron, and vitamin C contents. Furthermore, the blenderised ENF showed significantly higher levels (*p* < 0.05) of viscosity and osmolality than the commercial ENF. The significantly lower levels of some of the macro-nutrients and micro-nutrients in the blenderised ENF compared with the commercial ENF and the difference in the expected nutritional values may be due to the fact blenderised ENF is produced from common foods. Thus, the type of foodstuffs, cooking, and processing methods may lead to loss of nutrients and energy density. The deficits in the energy content and some of the macro- and micro-nutrients in the blenderised ENF compared with commercial ENF may have implications for patients’ health and clinical outcomes. The clinical implications of the underdelivering of nutrients may include increased risk of undernutrition, including energy malnutrition, which could have a negative effect on body composition and anthropometric parameters, morbidity, mortality, length of hospital stay, and costs. For outpatient care, this could increase the risk of hospital re-admission and homecare costs. Additionally, the higher viscosity and osmolality of the blenderised ENF compared with the commercial ENF can increase the risk of complications, including tube blockage, and impaired delivery of feed, water, and medications, with significant implications for patients’ nutritional status and health outcomes. Conclusion: The results of this systematic review and meta-analysis identified significant variability in the nutritional value of blenderised ENF compared with commercial ENF. Furthermore, the nutritional values of the blenderised ENF do not meet the expected recommended levels compared with commercial ENF and these may have implications for patients’ nutritional status and health outcomes, including the effect on body composition, morbidity, mortality, hospital re-admission, and costs. Further studies are needed to elucidate the nutritional value of blenderised ENF on patients’ clinical outcomes.

## 1. Introduction

Patients who are treated in hospital, long term facilities, or receive care at home, including those who are critically ill, are at risk of malnutrition [1,2]. The effect of malnutrition can be profound, with a higher rate of complications, such as increased length of hospital stay, and a higher risk of morbidity and mortality [2]. The provision of enteral nutrition is a useful way of mitigating these complications [1,3]. Enteral feed includes either a pre-packaged, ready-to-use formula or a powder formula which requires reconstitution, which can be delivered through enteral feeding tubes.

Enteral tube feeding is an effective method of providing nutritional support to individuals with a functional gastrointestinal tract who may be unable to meet their nutritional requirements through the oral route [2,4,5]. Individuals may be unable to meet their nutrition needs due to various diseases such as stroke, motor neurone disease, multiple sclerosis, dementia, head and neck cancer, and other conditions which could impair their swallowing ability [4]. Therefore, enteral tube feeding can be used for the management of malnutrition or can be a prophylactic measure to reduce the risk of malnutrition.

Due to the varied nature of the conditions that impact on nutritional intake and the requirements of individual patients, a range of nutritional formulas have been developed [3]. Although the history of enteral nutrition formula (ENF) can be traced to the use of homemade and/or blended ENF, the use of commercial ENF has been on the increase globally [3,6], especially in developed countries due partly to advances in technology and the increasing ageing population [7]. For example, patients receiving home enteral nutrition increased from 34,000 in 1989 to 344,000 in 2014 in the USA [6]. In the UK, the prevalence of home enteral tube feeding fluctuated between 2010 (*n* = 3430) and 2015 (*n* = 6270), with an overall increase of 10% since 2010 [8].

The main commercial ENF include standard polymeric, oligomeric, and monomeric diets (elemental formulas) and disease-specific formulas [3,6,9,10]. Despite the merits of using commercial ENF, including prevention of weight loss and reduced microbial risks compared with blended ENF [2,10,11,12,13], there is a growing preference to the use of blended ENF [3,14]. In some developing economies such as Iran, the use of blended ENF appears to be the primary enteral feed of choice and most hospitals are still using the traditional hospital prepared blended ENF [1,3]. The blended ENF is any food, whether liquid or food mixture, that is administered through an enteral feeding tube that is not water, medicine, and/or commercial ENF [6]. According to Brown [10], it is a homemade food that is blended to a smooth consistency.

The use of blended ENF may be due to the fact it is more affordable and improves reflux and bowel problems [12]. It has also been suggested that blended ENF are more natural, promote flexibility in the selection of ingredients, and enable patients to consume the same food as members of their family [6]. However, the use of blended ENF is limited by concerns relating to its nutritional value and physical and chemical instability compared with commercial ENF [2,6,12]. The main problems with blended ENF include the value of the nutrients, the viscosity of the formulas, and whether these formulas can indeed provide the daily nutritional requirements for the patients [2,6,12]. Therefore, the nutritional value of enteral nutrition formulas in the current review was based on the caloric density and the percentage of macronutrients and micronutrients to meet the UK and international dietary recommendations, although the nutritional quality of the enteral formula is also important [15,16]. The physical properties of blendrised ENF may be associated with adverse events such as tube blockage, diarrhoea, and constipation [6]. These limitations have implications for patients’ nutritional status and health outcomes and may explain why the British Dietetic Association currently does not recommend the use of a blended diet as a first choice in enteral tube feeding [10].

Therefore, it is necessary to evaluate the evidence concerning the use of blended ENF compared with commercial ENF as the strengths and limitations of these formulas are not consistent. While previous reviews have tended to focus on a critical review of this topic [3], integrative review [17], and reviews involving children [18], there appears to be no evidence drawn from a meta-analysis on the nutritional value of blended diets in the adult population. The present review is a systematic review and meta-analysis that aims to compare the nutritional value, physical properties, and clinical outcomes of blended ENF with commercial ENF.

## 2. Methods

The preferred reporting items for systematic reviews and meta-analyses (PRISMA) [19] was the method used for this review.

### 2.1. Study Designs and Samples

Experimental designs, including case-control, cross-sectional, and cohort studies, were included in this review and the samples were blended diets or blenderised ENF and commercial ENF.

### 2.2. Type of Population

Any person over the age of 18, with any type of disease that required enteral feeding. 

### 2.3. Type of Intervention and Comparison 

Blended versus commercial ENF.

### 2.4. Types of Outcome

The outcome measures of interest include;

- Nutritional value of enteral nutrition formulas—Energy, Macronutrients (protein, carbohydrate, fat), Micronutrients (sodium, potassium, calcium, phosphorus, magnesium, zinc, iron, vitamin A, vitamin C)
- Physical properties—Viscosity, osmolality
- Any type of clinical outcomes
- Adverse events—diarrhoea, tube blockage

### 2.5. Inclusion and Exclusion Criteria

The inclusion and exclusion criteria used to select studies for this review were based on the Population, Intervention, Comparator, Outcomes (PICO) framework [20].

Inclusion Criteria: Studies involving the population or problem of interest that included patients (adults) on blenderised ENF or blended tube feeding (BTF) were selected for this review. The intervention criteria were blended or blenderised ENF, while the comparator was the commercial ENF. The outcomes of interest for selecting studies were nutritional value, physical property, clinical outcomes, and adverse events. The studies included in this review were quantitative studies with a comparison group.

Exclusion Criteria: Studies involving children aged below 18 years and individuals on normal oral dietary intake were excluded from the review. Furthermore, patients on parenteral nutrition, parenteral plus enteral nutrition, and studies involving qualitative outcomes, such as patients’ feelings, were excluded from the review.

### 2.6. Search Strategy

The following databases; Pubmed, EMBASE, PSYCInfo, and Google scholar were searched for articles of interest using keywords, Medical Subject Heading (MeSH) and Boolean operators (AND/OR). The searches were carried out from the inception of each database until 23rd February 2020 and included the following keywords and combinations; Patients on blended tube feeding OR blended tube feeding AND blended tube feeding OR blenderised tube feeding OR blenderised enteral formula OR Homemade blenderised food OR blenderised diet OR blenderised feed OR blended formula. The search strategy was based on the Population or Problem, Intervention, Comparator, Outcomes—PICO framework [20].

**Figure 1:**
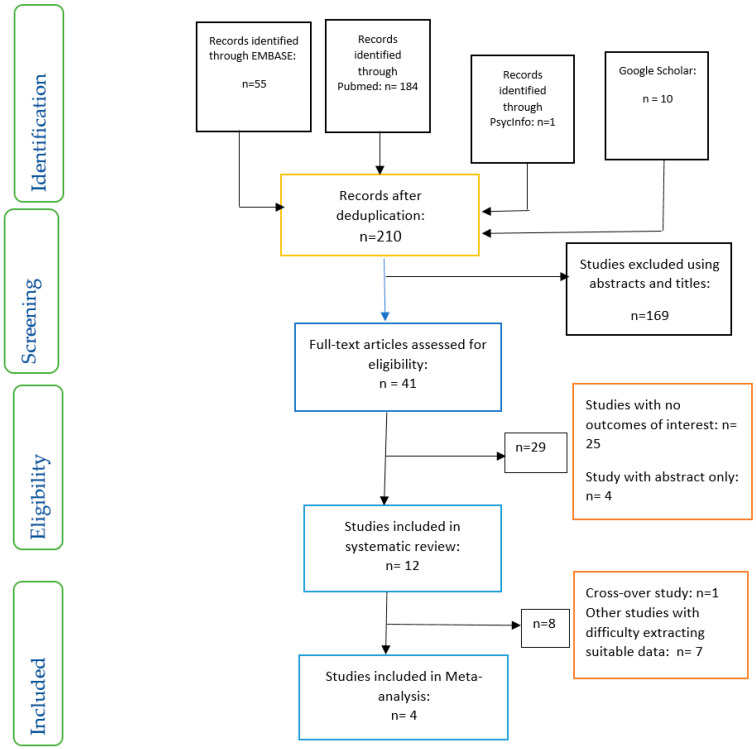
Preferred reporting items for systematic reviews and meta-analyses (PRISMA) flow chart on selection and inclusion of studies.

The studies reviewed were screened and evaluated for eligibility for inclusion based on the PRISMA guidelines [19] (Figure 1). This search and selection process of articles was conducted by two researchers (OO, OOO) and the resolution of differences was by consensus.

### 2.7. Data Extraction

The articles retrieved from the databases were exported to ENDNote (Analytics, Philadelphia, PA, USA) and duplicates were removed. The data from the included studies were extracted by one researcher (OO) and cross-checked by the other four researchers (OOO, AARA, X-HW, JB).

### 2.8. Statistical Analysis

The data extracted from the studies included in the review were analysed using RevMan (Review Manager, 5.3) [21]. The data analysis involved meta-analysis and sensitivity analysis. The sensitivity analysis was conducted by removing one study at a time (including studies with multiple sites and different blenderised ENF) from the meta-analysis to test for consistency of the effect of blended ENF on the parameters of interest. It was to check whether one particular study was having a profound effect on the results of the meta-analysis and how robust the differences were between the intervention and the control. Due to the varied nature of the studies included, the random-effects model was used for the meta-analysis and heterogeneity was measured by the statistic *I*^2^. A *p*-value of 0.10 was used to establish the statistical significance of heterogeneity.

### 2.9. Effect Size

The overall effect of the intervention in terms of statistical significance was determined by a *p*-value of <0.05 and the results of the meta-analysis were presented in the form of a forest plot.

## 3. Results

### 3.1. Managing the Data for Meta-Analysis

With respect to the Mokhalalati et el. [22] study, data from BTF samples (three samples over three days) from three different sites (hospitals) for the standard formula were combined, while data from three different sites (hospitals) for the therapeutic formulas were also combined and compared separately with samples of commercial ENF (sample size halved for each set of analysis) as recommended by Higgins and Green [23]. Therefore, for continuous outcomes in case of multiple arms, only the sample size was divided up and the means and standard deviations left unchanged. Data from commercial powder formulas (ensure powder and tap water in Hospitals A and B) were compared to blended ENF developed in Hospitals A and B, respectively, in the Sullivan et al. [24] study. On the other hand, the results of the commercial ENF (liquid and powder) were combined and compared with the homemade BTF in the Vieira et al. [25] study. The median and interquartile range (IQR) were converted to mean and standard deviation using a formulae proposed by Wan et al. [26].

Twelve studies were included in the systematic review including four studies included in the meta-analysis. Two studies each were conducted in Brazil [2,25], Iran [1,27], and the USA [6,28] and one study was conducted in each of the following countries (Table 1); Philippines [24], Poland [29], UK [12], Saudi Arabia [22], Thailand [30], and Greece [31]

### 3.2. Assessment of Risk of Bias and Evaluation of Quality

An appraisal of the quality of the articles was carried out using a critical appraisal skills programme (CASP) tool [32] and the Joanna Briggs Institute Critical Analysis Tool for Cross-sectional Studies [33] (Appendix A, Table A1 and Table A2). One researcher (JB) carried out the quality evaluation of the studies included and this was cross-checked by the other researchers. The data available in the studies were the only information used to assess the quality. Only studies involving human subjects where quality evaluation tools were available were assessed.

Based on the systematic review and meta-analysis, four distinct themes were identified; Nutritional value, Physical properties, Clinical outcomes, and Adverse events.

### 3.3. Nutritional Value of Blenderised Enteral Nutrtion Formulas 

In the studies conducted by Borghi et al. [2] and Mokhalalati et al. [22], the nutritional value of blenderised ENF were found to be highly variable and inconsistent (Table 1). Similarly, Sullivan et al. [24] hospital prepared BTF also demonstrated unpredictable levels of micronutrients and macronutrients and suggested that these diets may provide less than the required amounts of nutrients. In the Vieira et al. [25] study, energy and macronutrient values in the blenderised ENF were lower and provided less than 50% of the recommended values.

Jolfaie et al. [27] noted that commercial ENF contained more energy and nutrients compared with blended ENF.

The results of the meta-analysis demonstrated that there were no significant differences (*p* > 0.05) between the blenderised ENF and the commercial ENF in relation to the fat and protein contents of the diets (Table 2). However, the sensitivity analysis showed significant differences (*p* < 0.05) between these diets with respect to fat content when Sullivan et al. [24] blenderised ENF B was removed and for protein when Mokhalalati et al. [22] standard and therapeutic formulas were removed one at a time from the meta-analysis.

**Figure 2:**
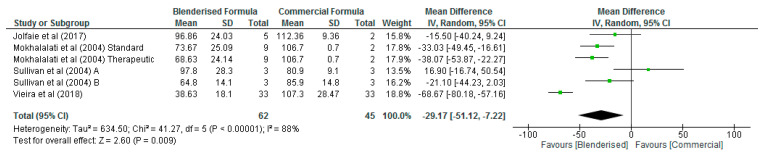
Shows energy content of blenderised and commercial ENF (Kcal/100 mL).

**Figure 3:**
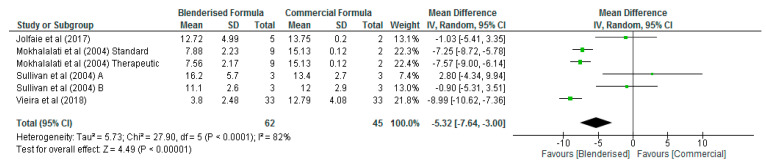
Shows carbohydrate content of blenderised and commercial ENF (g/100 mL).

However, the blenderised ENF was significantly lower (*p* = 0.009) than the commercial ENF with respect to the energy content of the diets with an overall mean difference of −29.17 Kcal/100 mL (95% CI, −51.12, −7.22) (Figure 2). Similarly, the carbohydrate content of the blenderised enteral formula was significantly lower (*p* < 0.001) than the commercial ENF, with an overall mean difference of −5.32 g/100 mL (95% CI, −7.64, −3.00) (Figure 3). The results of the sensitivity analysis also demonstrated significant differences (*p* < 0.05) between the blenderised ENF and the commercial ENF with respect to the energy density and carbohydrate content of the diets.

**Figure 4:**
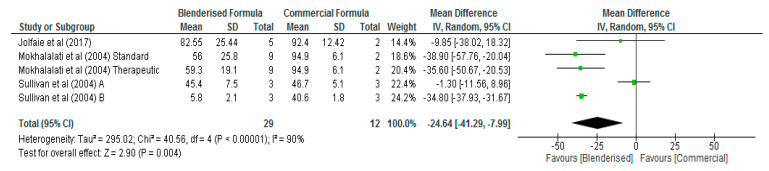
Shows calcium content of blenderised and commercial ENF (mg/100 mL).

**Figure 5:**
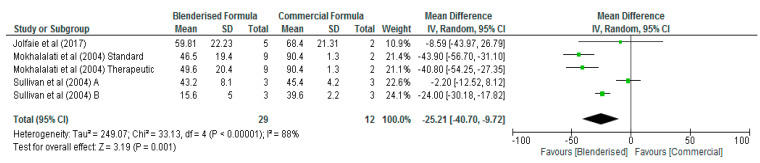
Shows phosphorus content of blenderised and commercial ENF (mg/100 mL).

**Figure 6:**
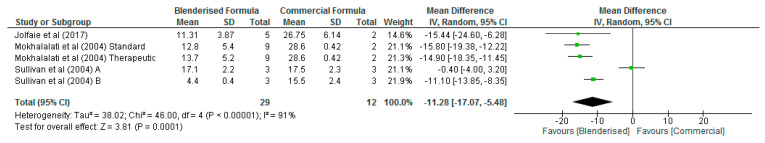
Shows magnesium content of blenderised and commercial ENF (mg/100 mL).

**Figure 7:**
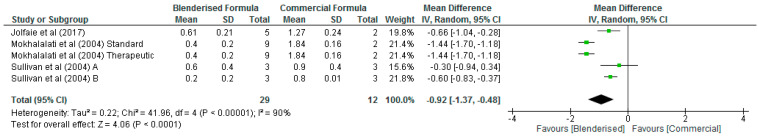
Shows zinc content of blenderised and commercial ENF (mg/100 mL).

**Figure 8:**
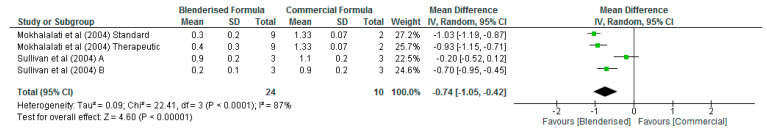
Shows iron content of blenderised and commercial ENF (mg/100 mL).

**Figure 9:**
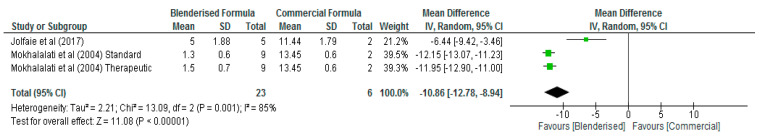
Shows vitamin C content of blenderised and commercial ENF (mg/100 mL).

In terms of sodium, potassium, and vitamin A, the results of the meta-analysis showed that the levels of these micronutrients were not significantly different (*p* > 0.05) between the blenderised and commercial ENF (Table 2). However, significant differences (*p* < 0.05) were observed between the two diets with respect to calcium, phosphorus, magnesium, zinc, iron, and vitamin C contents (Figure 4, Figure 5, Figure 6, Figure 7, Figure 8 and Figure 9, respectively). The overall mean differences showed that the blenderised ENF was significantly lower (*p* < 0.05) by −24.64 mg/100 mL (95% CI, −41.29, −7.99) with respect to calcium levels, −25.21 mg/100 mL (95% CI, −40.70, −9.72) for phosphorus, −11.28 mg/100 mL (95% CI, −17.07, −5.48) for magnesium, −0.92 mg/100 mL (95% CI, −1.37, −0.48) for zinc, −0.74 mg/100 mL (95% CI, −1.05, −0.42) for iron, and −10.86 mg/100 mL (95% CI, −12.78, −8.94) for vitamin C. Based on the sensitivity test, the results between the blenderised ENF and commercial ENF were also significantly different (*p* < 0.05) regarding calcium, phosphorus, magnesium, zinc, iron, and vitamin C contents.

### 3.4. Physical Properties of the Blenderised Formulas

According to Mokhalalati et al. [22], there was a high degree of variability in the physical properties of blenderised ENF. In addition, Sullivan et al. [24] noted that due to the viscosity of blenderised ENF, it may not be suitable to deliver these feeds through feeding tubes.

**Figure 10:**
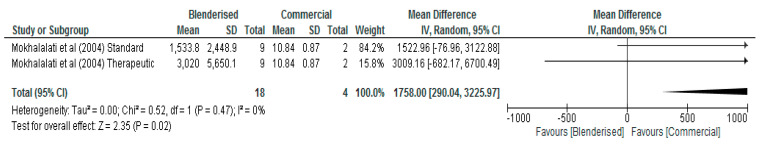
Shows the level of viscosity of blenderised and commercial ENF (Centipoise).

**Figure 11:**
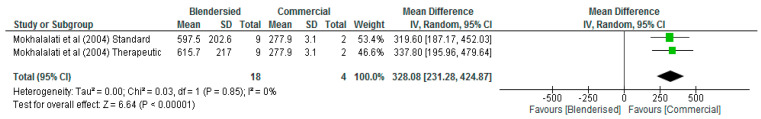
Shows osmolality of blenderised and commercial ENF (mOsm/kg H_2_O).

The results of the meta-analysis show that there are significant differences (*p* < 0.05) between the blenderised ENF and the commercial ENF in relation to viscosity and osmolality (Figure 10 and Figure 11, respectively). The blenderised ENF showed significantly higher levels (*p* < 0.05) of viscosity and osmolality with overall mean differences of 1758 Centipoise (95% CI, 290.04, 3225.97) and 328.08 mOsm/kg H_2_O (95% CI, 231.28, 424.87) for viscosity and osmolality, respectively.

### 3.5. Clinical Outcomes

According to Hurt et al. [6], most of the adult home enteral nutrition patients use BTF as part of their nutrition regimen during tube feeding and these patients did not report any significant concerns with BTF. Furthermore, Tiyapanjanit and Boonyavarakul [30] reported that the diabetic formula had significantly lower (*p* = 0.022) mean plasma glucose (122 ± 26.25 mg/dL) than the commercial diabetic formula (144.68 ± 36.91 mg/dL) and was also less expensive. In the hospital setting, Johnson et al. [28] revealed that BTF recipe selection and adherence to safe food handling provided a safe feeding that is comparable to commercial formula. 

However, evidence from the study by Jazayeri et al. [1] showed that there was increased macronutrient intake in the commercial ENF group and this was effective in promoting patients’ recovery compared with blenderised ENF. The authors concluded that the commercial ENF has more benefits than blenderised ENF in patients in intensive care unit. Klek et al. [29] also showed that the specialised home enteral tube feeding (HETF) care programme consisting of commercial ENF and a nutrition support team reduced morbidity and costs related to long-term enteral feeding at home.

While Papakostas et al. [31] found that blenderised foods do not adequately support the nutritional requirements of patients with head and neck cancer, Jolfaie et al. [27] observed that commercial ENF are more effective in meeting the nutritional requirements of patients who are fed enterally compared to blenderised ENF.

### 3.6. Adverse Events

According to Hurt et al. [6], about 12.5% of patients studied had concerns about the safety of blenderised ENF. However, 83.3% of the patients who used blenderised ENF did not experience nausea, vomiting, fever, or diarrhoea. In terms of tube blockages and time, while no blockages occurred in the standard commercial ENF irrespective of any tube diameter, two blockages occurred in the blendrised ENF A while using the 10Fr and 12 Fr tubes in the study by Madden et al. [12]. In addition, it was also significantly quicker to deliver the standard commercial ENF through the three tubes compared with the blended ENF [12].

## 4. Discussion

The findings of this review revealed inconsistencies in the macro-nutrient and micro-nutrient values of the blenderised ENF compared with the commercial formula. In addition, the nutritional value of the blenderised ENF did not meet all the expected nutrient standards compared with the commercial diet.

The results of the meta-analysis demonstrate that although there were no significant differences (*p* > 0.05) between blenderised ENF and commercial ENF in terms of fat, protein, sodium, potassium, and vitamin A contents, the differences were significant (*p* < 0.05) with respect to the energy density and other nutrients measured; carbohydrate, calcium, phosphorus, magnesium, zinc, iron, and vitamin C. Significantly higher (*p* < 0.05) levels of these nutrients were found in the commercial formula compared with the blenderised ENF. Furthermore, the results demonstrated that blenderised ENF had significantly higher (*p* < 0.05) levels of viscosity and osmolality than the commercial ENF and that the blenderised ENF also presented with a significant level of variability in their physical properties.

In relation to the clinical outcomes, the results of this review suggest that some of the patients may not be reporting significant concerns with the blenderised ENF [6]. According to Martin et al. [34], food based blendrised ENF provides a sense of normalcy for patients and caregivers of using regular foods. Furthermore, blenderised ENF can create a sense of enjoyment of meals, control of the foods that are used, and can boost the fibre content, phytonutrients, and pre-biotics in the diet [34]. However, challenges remain concerning deficits in the nutritional value, which may have implications for clinical outcomes. In addition, the delivery of blenderised ENF through feeding tubes may present significant challenges due to the high viscosity, osmolality, and increased risk of tube blockage in patients on enteral tube feeding.

The reduced macro-nutrient and micro-nutrient levels and energy density of blenderised ENF compared with commercial ENF is consistent with the Borghi et al. [2] study, which revealed energy density in blenderised ENF could range from 0.60–1.08 Kcal/mL, which represents low to normal energy density based on the European Society for clinical nutrition and metabolism (ESPEN) guidelines [35]. According to Lochs et al. [35], normal energy formulas should provide 0.9–1.2 Kcal/mL of energy. Energy levels in enteral feeds above this range are considered high, whereas energy density below the range is seen as low [35].

For standard enteral feed, which is suitable for most patients, the energy density should be 1 Kcal/mL with or without fibre [36]. On the other hand, high energy feed, which is usually used for patients on fluid restriction or increased nutritional requirements, should contain 1.2–2.0 Kcal/mL, while low energy formulas for patients with low energy requirements should have 0.5–1 Kcal/mL [36].

Mokhalalati et al. [22] found that the levels of vitamin A, sodium, and cholesterol in the blenderised ENF were significantly higher than the commercial ENF, although the levels of unsaturated fat, carbohydrate, energy, calcium, phosphorus, magnesium, zinc, iron, copper, and vitamin C were significantly lower for all the blenderised ENF compared with commercial ENF. Mokhalalati et al. [22] also observed that the blenderised ENF did not provide the predicted nutrient levels compared with the commercial ENF which demonstrated a high degree of accuracy in providing the expected nutrients with limited variability in nutritional values and physical properties. 

The nutritional value of blenderised ENF prepared from usual foodstuffs is often determined by the nutritional value of the foods used and these can be influenced by a range of factors including the geographical source of the food and the variety of the food [24]. Therefore, the significantly lower levels of some of the macro-nutrients and micro-nutrients in the blenderised ENF compared with the commercial ENF and the difference in the expected nutritional values may be due to the fact that blenderised ENF is produced from common foods such as milk, egg, meat, fruits, and vegetables which are cooked and pureed in a blender or mixer, often requiring sieving to remove large food particles [25]. Other factors that may lead to loss of nutrients could include the cooking method, processing method, the type of foodstuffs, stage of maturity of the crop used for food preparation, harvesting and storage methods, and human factors such as errors in measurements [22]. Another reason for the differences between the blenderised ENF and the commercial ENF is that there are no standard formulations for blenderised ENF preparations and the foodstuffs vary from setting to setting [27]. These factors may lead to the loss of a considerable amount of nutrients and energy in the feed [25]. The study by Madden et al. [12] also showed that after removing the waste (residues remaining on utensils and unsieved fraction), the remaining feed provided less than 95% of the estimated requirements for energy, fibre, iron, zinc, selenium, and vitamins A, D, E, and B_6_.

There is evidence that an institutionally prepared high-calorie formula did not provide the expected 1.5 Kcal/mL of energy; instead, it provided 1.0 Kcal and the formula did not meet the USA Recommended Dietary Allowance (RDA) for some of the minerals and vitamins [24]. In contrast, commercial ENF expected to provide 1.0 Kcal/mL and 1.5 Kcal/mL met all the nutrient and energy requirements [24]. It is essential that enteral formulas are well balanced in terms of their nutritional value, which is the basis for good health [37]. This is because the nutritional needs of patients are varied and often depend on their current and past nutritional status and the state of the complexity of their condition [38]. 

The implications of the nutritional differences between the blenderised ENF and the commercial ENF has public health significance because the patients who are fed the blenderised ENF may become deficient in energy, carbohydrate, and some of the micro-nutrients, including calcium, phosphorus, magnesium, zinc, iron, and vitamin C, as they do not meet the RDA for these nutrients compared with commercial ENF [2,24,25]. The adult RDA is seen as the average daily level of nutritional intake that is enough to meet the nutrient requirements of nearly all healthy people [39]. This standard is similar to the UK Public Health England [16] recommendations for energy, macro-nutrients, and micro-nutrients for different age groups including 19–64 years in the general population. In this regard, the daily energy intake recommended for males in this age group is 2500 Kcal/day and 2000 Kcal/day for women [16]. For carbohydrates, the recommended allowance is at least 333 g/day for males and 267 g/day for females in this age group [16]. While standard commercial ENF contain macro-nutrients and micro-nutrients that meet the standards recommended for healthy persons, it has also been shown that based on the caloric intake of a normal diet, the micro-nutrients supplied in commercial ENF are often above the Dietary Reference Values for healthy populations [3,37]. However, more energy and nutrients may be required in patients who are critically ill [3]. The calculation of the caloric requirements for patients is often based on equations such as the Harris-Benedict equation and indirect calorimetry for hospitalised patients, although the usual range is 25–30 Kcal/kg/day [38,40].

In order to provide the Recommended Dietary Intake for vitamins and minerals and reduce the risk of nutrient deficiency, the patient has to be fed 1200 to 1500 Kcal/day [15]. Iacone et al. [37] examined the micro-nutrient contents of 62 enteral nutrition formulas manufactured by five different companies at the doses of 1500 and 2000 Kcal/day and found that the daily requirements for all the micro-nutrients were covered, except for vitamin K and fluoride. The results of the present review are in agreement with these findings with respect to the micro-nutrient value of the commercial ENF. It is also clear that adequate macro-nutrients and micro-nutrients are necessary for the prevention and management of nutritional deficiency and the sustenance of normal metabolism [38].

The clinical implications of the underdelivering of nutrients may include adverse clinical outcomes such as accelerated loss of lean body mass [24]. In the study by Sullivan et al. [24], the mean calcium content obtained from 1000 Kcal for one of the blenderised ENF was 90 mg, while the US Dietary Recommended Reference Intake is 1000 mg calcium per day for adults in the 19–50 year group, and this is higher for patients who are at risk of osteoporosis.

The inadequacy in the energy and some of the nutrients in the blenderised ENF reduces the effectiveness of the nutritional therapy, which may increase the risk of undernutrition, including energy malnutrition, morbidity, mortality, length of hospital stay, and costs [2]. For outpatient care, this could increase the risk of hospital re-admission and homecare costs [2].

In a study by Vieira et al. [25], the patients that received blenderised ENF obtained less than 50% of the recommended values of nutrients and this had a significant impact on anthropometric and body composition parameters compared with those on commercial ENF. Furthermore, the study by Papakostas et al. [31] found that patients who consumed blenderised ENF during chemo-radiation therapy had a significant reduction in body mass index compared with those who had commercial ENF. In contrast, commercial ENF has been shown to improve patients’ weight and decrease infectious complications and hospital admissions [41].

The variations in the chemical composition, molecular sizes, solubility, and value of the macronutrients in ENF may influence the osmolarity, absorption, utilization of nutrients, and patients’ clinical outcomes [15]. Mokhalalati et al. [22] noted that the mean viscosity for the blenderised ENF was 2276.9 Centipoise (cP), compared with 10.84 cP for the commercial ENF, and for the commercial ENF that are fed unaided through small diameter enteral feeding tubes, such as 8Fr, the viscosity is usually less than 60 cP. This was a 200-fold higher level of viscosity and a two-fold higher level of osmolality in the blenderised ENF compared with commercial ENF [22]. The higher viscosity in the blenderised ENF could suggest that it will be difficult for some of the samples to flow easily through nasogastric and other enteral feeding tubes with a small diameter [24].

Therefore, the higher viscosity and osmolality of the blenderised ENF compared with the commercial ENF can increase the risk of complications including tube blockage and impaired delivery of feed, water, and medications, with significant implications for patients’ nutritional status and health outcomes.

### Limitations

The number of studies included in the meta-analysis was only four and this may affect its wider application. Due to the limited number of studies investigating clinical outcomes and the varied nature of the parameters measured by the studies, the data were insufficient to run a meta-analysis and present the forest plot for clinical outcomes. In addition, the lack of a quality evaluation tool to assess the in-vitro experimental studies and the conversion of the median and interquartile range to mean and standard deviation are potential limitations in this review. 

## 5. Conclusions

The results of this systematic review and meta-analysis identified significant variability in the nutritional value of blenderised ENF compared with commercial ENF. Additionally, the nutritional values of the blenderised ENF do not meet the expected recommended levels compared with commercial ENF and these may have implications for patients’ nutritional status and health outcomes, including the effect on body composition, morbidity, mortality, hospital re-admission, and costs. Further studies are needed to elucidate the nutritional value of blenderised ENF on patients’ clinical outcomes.

## Figures and Tables

**Table 1:** Shows results of the data extracted from the studies included.

Citation	Country	Aim/Objective of Study	Study Design	Study Method/Sample Size/Description	Age (Years)	STUDY Results/Conclusion
Jazayeri et al. [1]	Iran	To evaluate the effects of standard enteral feeding compared with hospital-prepared blended formula among Intensive Care Unit (ICU) patients.	Case-control study	A total of 80 patients were involved in the study. These included 40 patients in a standard enteral feeding group and another 40 patients in the hospital-prepared blended formula group.	Hospital-prepared blended formulas: 50.1 ± 18.7 Years Standard enteral feeding: 49.9 ± 18.1 Years	There was increased macronutrient intake in the standard ENF group and this helped in patients’ recovery. The standard enteral ENF has more benefits than hospital-prepared blended ENF for ICU patients.
Borghi et al. [2]	Brazil	To evaluate the nutritional quality and cost of blenderised tube diets (BTD).	In-vitro experimental study	Only five BTD out of 14 collected BDT recipes were analysed for their nutritional properties while the commercial foods were based on portion size and manufacturer’s information	Not Applicable	Blenderised tube feeding diets were highly variable and with inconsistent nutritional value.
Hurt et al. [6]	USA	To determine the prevalence and use of BTF and frequency of use in adults receiving HEN.	Cross-sectional study	All patients who had follow-up appointments at the HEN clinic were approached during their appointment to participate in completing the survey electronically or fill in a paper questionnaire. The survey consisted of 15 questions.	60.5 Years	Most of the adult HEN patients use BTF as part of their nutrition regimen during tube feeding. Patients did not report any significant concerns with BTF.
Madden et al. [12]	UK	To examine the risks of blended formula providing nutritionally adequate intake.	In-vitro experimental study	A blended formula was made using three different methods (professional, jug, and stick blenders) and three storage procedures. The feed samples were delivered through 10-, 12-, and 14-French (Fr) enteral feeding tubes and both blockages and the time taken were recorded.	Not Applicable	There was no risk of tube blockages when one blended ENF recipe made using three methods was delivered via a 14-Fr tube. After removing the waste (residues remaining on utensils and unsieved fraction), the remaining feed provided less than 95% of the estimated requirements for energy, fibre, iron, zinc, selenium, and vitamins A, D, E, and B_6_
Mokhalalati et al. [22]	Saudi Arabia	To compare the microbial safety, nutritional content, and physical properties of BTF and commercially prepared formulas (CPF).	In-vitro experimental study	18 samples of BTF were collected from 3 hospitals. Samples of a CPF were also collected for comparison.		There is a high degree of variability in nutrient content and physical properties of BTF. Cholesterol, sodium, vitamin A, and vitamin B_6_ levels for all BTF were higher than the commercial ENF. However, the values for unsaturated fat, nonferrous extract (NFE), calories, calcium, phosphorus, magnesium, zinc, iron, copper, and vitamins D, E, B_3_, and C were lower for all BTF compared with commercial ENF.
Sullivan et al. [24]	Philippines	To evaluate the nutritional content and viscosity of hospital-prepared BTF.	In-vitro experimental study	Two different BTFs (one standard and one modified) were collected from each hospital on three separate occasions and analysed for macronutrients, micronutrients, and viscosity.	Not Applicable	Hospital prepared BTF showed unpredictable levels of micronutrients and macronutrients and may provide less than the required amounts of nutrients. In addition, the viscosity of these formulas may not be suitable for infusion through feeding tubes.
Vieira et al. [25]	Brazil	To evaluate the nutritional and microbiological quality of commercial enteral and homemade blenderised whole foods.	Cross-sectional study	66 samples of commercial (*n* = 33) and noncommercial (*n* = 33) enteral diets were collected at the homes of patients on HEN	73 Years (20–100 Years)	The homemade blenderised ENF demonstrated low values of energy and macronutrients and provided less than 50% of the recommended values.
Jolfaie et al. [27]	Iran	To compare the nutritional quality of commercial enteral nutrition and blenderised enteral formula	Cross-sectional study	150 patients were fed blended formula and 120 patients were fed commercial ENF	Blenderised formula: 55.46 ± 20.19 Years Commercial Formula: 53.13 ± 20.35 Years	Commercial ENF contained more energy and nutrients compared with blended ENF and they are more effective in meeting the nutritional requirements of patients who are fed enterally.
Johnson et al. [28]	USA	To compare microbial loads of a standard polymeric commercial formula (CF), a BTF made using baby food (BTF-BF), and a BTF prepared from blending whole food (BTF-WF).	In-vitro experimental study	Three tube-feeding formulas (CF, BTF-BF, BTF-WF) were compared.	Not Applicable	The results show that BTF recipe selection and adherence to safe food handling provide a safe feeding that is comparable to CF in the hospital setting.
Klek et al. [29]	Poland	To examine the effect of commercial enteral nutrition (specialised home enteral nutrition) programme on clinical outcomes.	Cohort	All patients who had received home enteral tube feeding (HETF) with homemade blenderised diets for 12 months before starting a specialized nutrition programme for another 12 months consisting of the provision of commercial enteral nutrition formulas and guidance on nutrition support.	52.5 Years	It was demonstrated that the specialized HETF care programme consisting of commercial ENF and nutrition support team reduces morbidity and costs related to long-term enteral feeding at home.
Tiyapanjanit & Boonyavarakul [30]	Thailand	To compare blood glucose parameters and cost between the Phramongkutklao’s diabetic formula and commercial diabetic formula in patients with type 2 diabetes.	Cross-over study	Participants were fed using 24 h continuous feeding for three days. The Phramongkutklao’s diabetic formula was followed by commercial diabetic formula continuously for 36 h each.	79.80 ± 11.03 Years	The Phramongkutklao’s diabetic formula had significantly lower mean plasma glucose and was less expensive than the commercial diabetic formula.
Papakostas et al. [31]	Greece	To evaluate the body composition characteristics and nutritional status in HNC patients who are receiving either the prescribed commercial enteral nutrition formula or decided on home-made BTF	Quasi-experimental design	All patients were prescribed to receive, on an out-patient basis, a commercially available enteral nutrition formula. Patients with low income and no public health insurance were recommended to have equivalent home-made enteral formula. Both groups were also advised to consume, yogurt with honey, ice-cream, and fruit and vegetables. 212 patients including 112 who received the commercial formula, 69 who switched to BTF, and 31 that were prescribed to receive a home-made formula of standard ingredients were involved in the study.	Commercial: 56.4 ± 3.6 Years Home-made: 55.9 ± 3.5 Years Blenderised Family Food: 56.2 ± 3.8 Years	The results show that home-made and blenderised foods do not adequately support the nutritional requirements of patients with HNC.

Abbreviations: Blenderised tube diets (BTD); Blenderised tube feeding (BTF); Enteral Nutrition Formulas (ENF); Home enteral nutrition (HEN); Home enteral tube feeding (HETF); Head & Neck cancer (HNC).

**Table 2:** Shows the results of the meta-analysis of macro- and micro-nutrients in blenderised and commercial ENF.

Outcomes	Number of Studies/Experiments	Number of Samples	Mean Difference (95% CI)	*p*-Value	*I*^2^ %
Fat (g/100 mL)	6	107	−0.63 [−1.41, 0.14]	0.11	74
Protein (g/100 mL)	6	107	−0.76 [−1.64, 0.12]	0.09	76
Sodium (mg/100 mL)	5	41	−29.22 [−65.90, 7.46]	0.12	81
Potassium (mg/100 mL)	5	41	−27.68 [−74.88, 19.53]	0.25	88
Vitamin A (mcg/100 mL)	4	34	−2.03 [−37.73, 33.68]	0.91	82

## Appendix A

**Table A1:** CASP Critical Analysis Tool for cohort studies.

	Klek et al. [29]	Jazayeri et al. [1]	Tiyapanjanit et al. [30]	Papakostas et al. [31]
Section A				
1. Did the study address a clearly focused issue?	YES	YES	YES	YES
2. Was the cohort recruited in an acceptable way?	YES	YES	YES	YES
3. Was the exposure accurately measured to minimise bias?	YES	YES	YES	YES
4. Was the outcome accurately measured to minimise bias?	YES	YES	YES	YES
5a. Have the authors identified all important confounding factors?	YES	YES	YES	YES
5b. Have they taken account of the confounding factors in the design and analysis?	YES	YES	YES	YES
6a. Was the follow up of subjects complete enough?	YES	YES	YES	YES
6b. Was the follow up of subjects long enough?	YES	YES	Unclear why the first and third day of enteral feeding were monitored	YES
Section B				
7. What are the results of the study?	SpecializedHETF care program reduces morbidity and costs related to long-term enteral feeding at home.	Albumin levels were significantly increased in the manufactured standard enteral formula compared to the hospital prepared blended formula.	Hospital blenderised formula resulted in significantly lower mean plasma glucose, cost, and number of capillary blood glucose tests, compared to commercial diabetic formula.	Commercially available, oncology specific enteral feeding formulas have significantly better nutritional profiles than the blenderised home-cooked diets.
8. How precise are the results?	The results were significant and presented the CIs, demonstrating reasonable precision	Significant results were presented	Significant results were presented	Significant results were presented
9. Do you believe the results?	YES	YES	YES	YES
Section C				
10. Can the results be applied to the local population?	YES	YES	NO—sample of 10 participants	YES
11. Do the results of this study fit with other available evidence?	YES	Unclear	NO	Unclear
12. What are the implications of this study?	Patients receiving home enteral nutrition should be adequately supervised and provided with appropriate enteral diets to maximise the benefits of such a therapy.	Standard enteral formula has more benefits than hospital-prepared blended formulas for ICU patients.	The hospital diabetic formula had significantly lower mean plasma glucose and was less expensive than the commercial diabetic formula.	Home-made and family-cooked food is inappropriate and may actually be harmful for malnourished HNC patients scheduled to receive concurrent treatment.

Abbreviations: CASP (critical appraisal skills programme); ICU (Intensive Care Unit); Homet enteral tube feeding (HETF); Confidence Intervals (CIs).

**Table A2:** Joanna Briggs Institute Critical Analysis Tool for Cross-sectional Studies.

	Hurt et al. [6]	Vieira et al. [25]	Jolfaie et al. [27]
1. Were the criteria for inclusion in the sample clearly defined?	YES	YES	YES
2. Were the study subjects and the setting described in detail?	YES	YES—commercial and non-commercial enteral feeds	YES—both participants and commercial and non-commercial formulas
3. Was the exposure measured in a valid and reliable way?	YES	NOT APPLICALBE	YES
4. Were objective, standard criteria used for measurement of the condition?	YES—received commercial enteral nutrition for 3 weeks	YES—for each commercial and non-commercial enteral feeds	YES—for commercial and non-commercial formulas
5. Were confounding factors identified?	YES	NO	YES
6. Were strategies to deal with confounding factors stated?	NOT APPLICABLE	NO	YES
7. Were the outcomes measured in a valid and reliable way?	YES—developed and validated a survey, but analysis not provided	YES—the content of the feeds	YES—the content of the feeds
8. Was appropriate statistical analysis used?	YES	YES	YES
